# Protocol for the rural engagement in TelemedTeam for options in obesity treatment solutions (RE-TOOL): Cluster randomized trial investigating team-based telemedicine in rural primary care

**DOI:** 10.1016/j.conctc.2025.101559

**Published:** 2025-10-11

**Authors:** Alexandra R. Brown, Edward F. Ellerbeck, Debra K. Sullivan, Eve-Lynn Nelson, Jennifer R. Klemp, Byron J. Gajewski, Jarron Michael Saint Onge, Christie A. Befort

**Affiliations:** a Department of Biostatistics & Data Science, University of Kansas Medical Center, 3901 Rainbow Blvd, MS 1026, Kansas City, KS, 66160, USA; b Department of Population Health, University of Kansas Medical Center, 3901 Rainbow Blvd, MS 1008, Kansas City, KS, 66160, USA; c Department of Dietetics and Nutrition, University of Kansas Medical Center, 3901 Rainbow Blvd, MS 4013, Kansas City, KS, 66160, USA; d Department of Pediatrics, University of Kansas Medical Center, 3901 Rainbow Blvd, MS 4004, Kansas City, KS, 66160, USA; e Division of Medical Oncology, University of Kansas Medical Center, 3901 Rainbow Blvd, MS 1022, Kansas City, KS, 66160, USA

**Keywords:** Obesity, Rural, Behavioral weight loss, Primary care, Telemedicine, Randomized clinical trial

## Abstract

Rural communities experience disproportionately high rates of obesity and related chronic diseases. Rural residents report a lack of weight control programs within their communities, leaving primary care physicians (PCPs)at the center of obesity treatment. PCP involvement significantly enhances uptake and maintenance of weight loss behaviors, but PCPs face significant challenges in delivering consistent, high quality obesity treatment. Capitalizing on the rapid expansion of telehealth, this cluster-randomized trial will evaluate the effectiveness of a novel team-based treatment approach in reducing weight compared to standard quarterly PCP visits. Team Care is a telemedicine approach that pairs intensive telemedicine group visits with quarterly individual team-based clinic visits that simultaneously engage the participant, the local PCP, and a lifestyle coach. This combines the benefits of group-based treatment with home-based telemedicine delivery, and critically, integrates team-based care in local rural clinics. We hypothesize that the team-based approach will be more effective in achieving weight loss at 18 months. Sixteen practices from rural Kansas will be randomized to deliver the team-based approach or standard of care to 35 participants per practice (n = 560) age 20 to 75 with a BMI at least 30 kg/m2. Secondary endpoints include clinical cut points for weight loss, quality of life indicators, and implementation process measures. This research will advance knowledge of obesity treatment in rural primary care by directly comparing the effectiveness of an alternative model of care with the current standard of care. The results may warrant a new standard of care for obesity treatment in rural primary care practices.

## 1 Introduction

Obesity now affects over 40 % of the U.S. adult population [1] with the prevalence of obesity among rural Americans being even higher [2,3]. Most concerning, the prevalence of class 3 obesity (BMI ≥40), which contributes the highest cancer and other chronic disease risk [4], is increasing at a rate 3 times higher in rural communities compared to urban areas [3]. Rural residents will continue to experience significantly higher excess deaths unless something is done to stop this widening rural-urban obesity disparity [5].

Rural residents experience unique barriers to maintaining a healthy lifestyle, including lack of access to weight control programs in their communities, especially in small or remote rural areas [[6], [7], [8], [9]]. Moreover, rural communities have fewer environmental resources that promote healthy lifestyles [10], with individuals walking less for transportation or exercise [11] and consuming higher-fat and lower plant-based diets [2,12], that may lead to normative health compromising behaviors particularly among those with lower socioeconomic status [[13], [14], [15]].

PCP involvement significantly enhances the uptake and maintenance of weight loss behaviors [16,17]. It is essential to address co-morbid medical conditions and medications that can impede weight loss [18], and evaluate for guideline-recommended medical and surgical treatment options. Hence it is paramount that treatment be offered in rural primary care reaching those who have Class 3 obesity and/or have co-morbid medical conditions that can increase obesity-related morbidity and mortality [[19], [20], [21]]. There has been a missed opportunity, however, to integrate PCP medical management with behavioral weight loss.

Capitalizing on the expanded capacity for home-based telemedicine [22] and lessons learned in our previous study [23], we will conduct **RE-TOOL** (**R**ural **E**ngagement in **T**elemedTeam for **O**ptions in **O**besity Treatment So**L**utions). This 5-year cluster randomized trial will compare a novel team-based telemedicine approach*,* that pairs intensive group telemedicine visits with a lifestyle coach, to enhanced standard of care in rural primary care practices. We hypothesize the team-based telemedicine approach (Team Care) will be more effective compared to enhanced usual care (Local Care) in maximizing percent weight loss at 18 months.

## 2 Methods

### 2.1 Overview

**Fig. 1:**
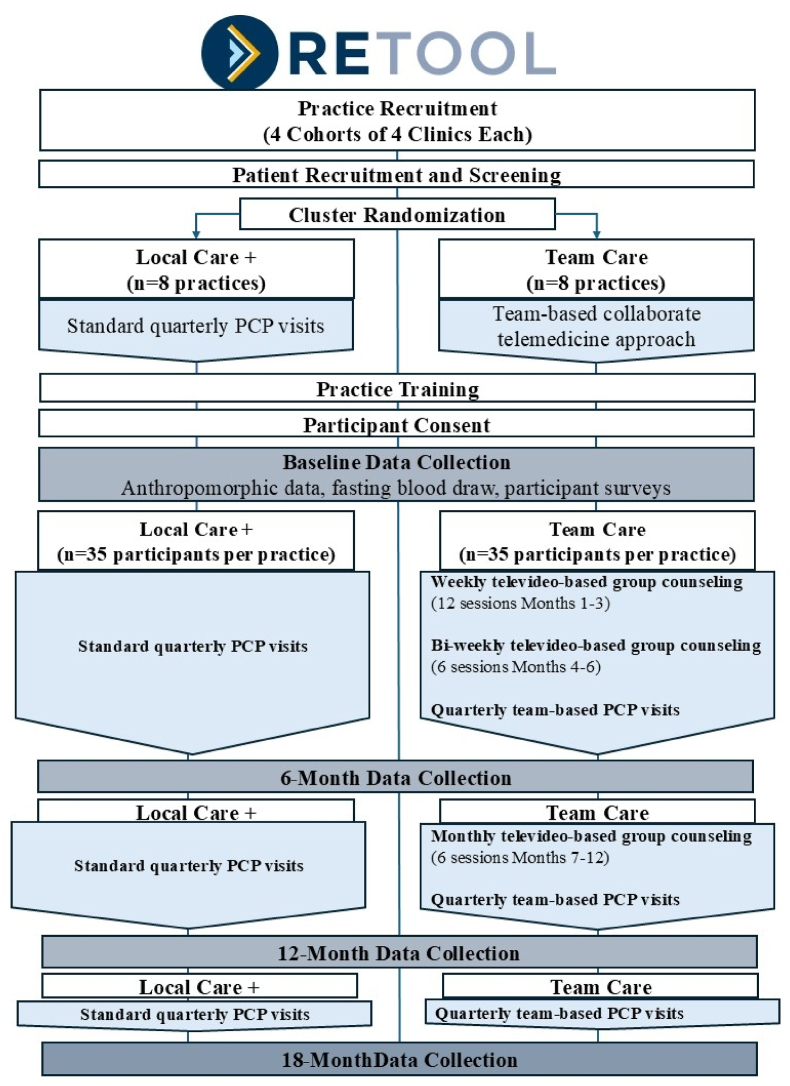
RE-TOOL study flow chart.

Sixteen rural primary care clinics will be randomized in equal proportions (1:1) to one of two study arms (Team Care vs. Local Care) to deliver obesity treatment to eligible participants. Each clinic will enroll approximately 35 participants and retain them over 18 months. The primary outcome is percent weight loss at 18 months. Fig. 1 provides an overview of the study flow diagram.

### 2.2 Aims

The primary aim of the study aims to evaluate the hypothesis that the Team Care model will result in greater % weight loss across 18 months compared to the Local Care + arm. Secondary aims will examine whether Team Care leads to:

1. Higher rates of achieving ≥5 % and ≥10 % weight loss at 6, 12, and 18 months.
2. Improved outcomes in weight (kg), diet quality, physical activity, and quality of life across 6, 12, and 18 months.

Tertiary aims will compare the effects of the two treatments on mental health, pain, sleep quality, and medical treatment process outcomes (participant activation, participant-provider communication, discussion and prescription of weight loss medications, and discussion and referral for bariatric surgery) across the study. We will also evaluate the uptake of pharmacotherapy and bariatric surgery and their potential impact on the between-arm difference in % weight loss. Exploratory aims will evaluate reach, adoption, implementation, and maintenance of the intervention according to the RE-AIM evaluation framework [23]; and to investigate rural sociocultural and spatial predictors of treatment effects.

### 2.3 Primary care practice and provider recruitment

Clinics intend to be recruited through connections facilitated by the study team and Community Advisory Board. Rural primary care practices that predominately or exclusively serve rural residents in Kansas will be enrolled. A total of sixteen clinics in 4 cohorts of 4, with different degrees of rurality and region, are planned to be recruited.

All practices will be required to identify a PCP champion and a primary administrative contact. Practices must be able to provide a registry of 300–400 patients with a BMI ≥30 kg/m2 in accordance with their practice's HIPAA policies. During the practice recruitment process, the PCP champion will approach other providers at the practice to offer the opportunity to participate in the study. Participating providers must be willing to provide enrolled patients at least quarterly visits for weight management, and if randomized to Team Care, to engage with the lifestyle coaches via telemedicine during those quarterly clinic visits.

### 2.4 Cluster randomization

Primary care practices will be randomly allocated in equal proportions (1:1) to each of the two study arms (Team Care, Local Care) in pairs after the target number of participants at the sites have been screened eligible and eligible participants have completed their initial baseline surveys. Eight practices are expected to enroll into each arm. Participants remain blinded to randomization until fully enrolled in the study. Practice staff will be notified during final participant enrollment steps to allow for sufficient lead time for practice staff to schedule the first quarterly clinic visits approximate in time to the baseline weight measurement.

### 2.5 Participant inclusion and exclusion criteria

Patients at participating rural clinics will be eligible if they are between the ages of 20.0 and 75.0, have a BMI at least 30.0 kg/m^2^, and reside in a rural location as defined by Rural-Urban Commuting Area Codes [24], Urban Influence Codes [25], amount of agricultural income, or individual commuting patterns. Participants must speak English, have provider clearance to participate, be able to walk without assistance, have been seen at least once in the participating clinic within the past year, and have internet access or a smart phone. Participants will be excluded for the following reasons: history of myocardial infarction, stroke, or new cancer diagnosis within the last six months; history of bariatric surgery within the last two years; pregnancy in the last 6 months or planned within the next 18 months; currently lactating; end stage renal or liver disease or anticipated renal or liver transplant within the next 18 months; currently on or anticipated dialysis in the next 18 months; currently enrolled or planning to enroll in another study where weight loss is targeted; currently planning to move outside of current provider area or leave primary care clinic within the next 18 months. Only one individual per household is permitted to enroll in the study.

### 2.6 Participant recruitment

Participants will be recruited from the registry of patients from the 16 participating rural primary care practices using both mail and in-clinic referrals following the methods used in RE-POWER, which resulted in timely recruitment, a high enrollment rate (86 %), and a study sample that was largely representative of the broader clinic populations with a high level of co-morbidity [26]. On behalf of the practice, the study team will send recruitment letters to registry patients and include a study invitation letter signed by the local PCP, a study brochure, and a pre-stamped opt-in postcard. Recruitment materials will refer participants to the study team, their local clinics, or the study website for more information. PCPs will also directly refer patients during routine medical visits (active approach), and recruitment cards and brochures are distributed in the clinics (passive approach).

### 2.7 Screening and informed consent

Study staff intends to contact potential participants via telephone to inform them about the study, assess eligibility, and gauge interest. Following screening, clearance to participate from the PCP is obtained and participants will be sent an initial baseline survey packet either by mail or online link. When enough participants complete the initial surveys, the practice will be randomized to a treatment arm and participants will be sent a second survey packet. Study staff will contact the participant to schedule an e-Consent interview upon completion of the second survey packet. Once consented, they will be scheduled for their Zoom baseline data collection visit for the weight measure. To reach full enrollment in the trial, the participant must complete the baseline data collection visit.

### 2.8 Intervention descriptions

**Fig. 2:**
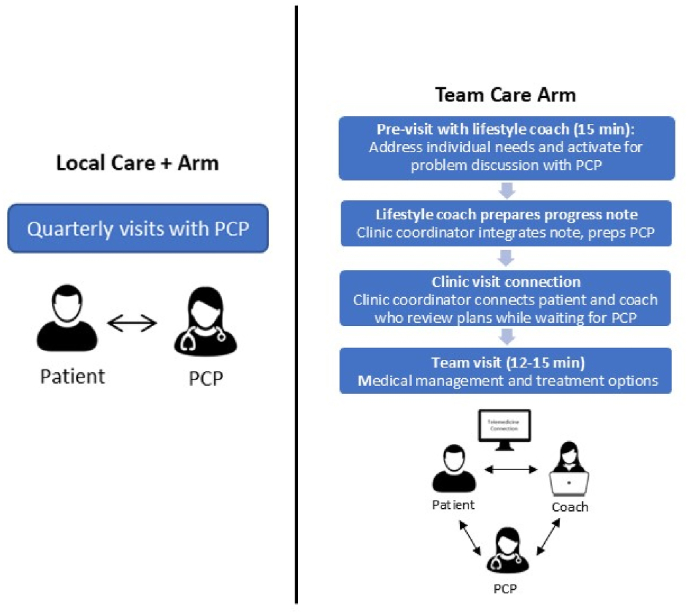
Quarterly visit comparison by arm.

*Team Care.* Team Care will include a group telemedicine lifestyle intervention plus quarterly telemedicine individual visits with the patient and lifestyle coach followed by quarterly clinic team visits with the patient, lifestyle coach, and PCP together (see Fig. 2).

Team visits. Team Care PCPs address medical management of obesity with lifestyle coaches joining the visit via zoom on an iPad. Team-based care is accomplished by both the PCP and the coach 1) understanding the scope of each other's role, 2) providing the patient with consistent messaging, and 3) fostering on-going open communication [27]. The PCP's role focuses on integration of medical management whereas the coach's role focused on lifestyle intervention and continuity of care (see Table 1). Approximately one week prior to the PCP visit, the lifestyle coach (the same coach who leads the group intervention) will meet with the patient via zoom for an individual ‘pre-visit.’ In addition to activating the patient for problem discussion with their PCP, these visits address individual needs related to lifestyle change which can be particularly important during weight loss maintenance and also can help facilitate more meaningful engagement in the group intervention. The lifestyle coach will share a progress report with the PCP summarizing the patient's weight history and current weight loss, adherence to nutrition and physical activity goals, known barriers, and medical concerns for discussion including patient's current perceptions about anti-obesity medications or surgical options. The PCP will review the note immediately prior to the team visit. During the team visit, progress and barriers toward lifestyle change will be discussed, medication preferences and options will be discussed, and alignment on the treatment plan is developed. Depending on the preferences of the patient and PCP, the coach may drop off during the visit if non-obesity treatment plans need to be addressed.

**Table 1:** Roles during Team Clinic visits.

**Role: Lifestyle coach**
• Provide intensive lifestyle intervention. • Provide guidance, feedback, accountability toward nutrition and PA changes. • Provide continuity of care before, during, after team visits. • Activate and prepare patient to get the most out of clinic visits (prompt for concerns, questions). • Resource for follow-up. • Can drop off Zoom at any time if PCP and patient have other issues to cover.
**Role: PCP**
• Integration of obesity management into overall medical care. • Communicate benefits of weight loss with comorbid conditions, labs, meds. • Monitor meds for co-morbid conditions: weight neutral prescribing and other adjustments as needed. • Prescribe anti-obesity medications if indicated. • Specialty referrals as needed: • Bariatric surgery • PT or Dietetics • Mental health.

Group-based comprehensive lifestyle intervention. The lifestyle intervention will be modeled after the Diabetes Prevention Program [28] and the Look AHEAD Lifestyle Intervention [29] and is guided by a social-cognitive framework [30]. Groups include approximately 12 participants with visits lasting 60 min weekly for 12 weeks, bi-weekly for 12 weeks, and monthly for 12 months. The intervention will be targeted to the rural setting in the Midwestern region (Table 2) and will highlight shared rural social identity while at the same time attending to differences that exist. Each participant will receive a treatment handbook with session-by-session content.

**Table 2:** Rural targeted intervention components.

Simplified educational materials, self-monitoring forms to address lower education levels
Recipes of low-fat, high FV versions of traditional “country” or “potluck” dishes
Problem-solving re: barriers to accessing PA facilities and healthy foods in grocery stores and restaurants
Alternative home-based activities when walking is not feasible due to weather/environmental constraints
Linking behavior change to cultural values related to hard work and family priorities

During the weight loss phase, participants will receive instructions to follow a reduced-calorie diet and will be asked to track their food intake through the MyNetDiary [31] app that syncs with a study-provided Fitbit and smart scale. Coaches plan to review food logs, physical activity, and weights and provide feedback within the app focusing on progress toward participants weekly goals. Throughout the program, an emphasis will be placed on learning meal planning and preparation, attending to portion control, and consuming at least 5 fruit and vegetable (FV) servings per day, lean proteins, and whole grains [32]. To jump start weight loss, initial dietary recommendations include a calorie range goal, 2–3 protein shakes per day, and 2 prepackaged meals per day. Participants will purchase their own food. After week 6, protein shakes and prepackaged meals will be recommended for breakfast and lunch only along with a home-prepared supper meal. After week 12, participants will be instructed to plan and prepare 3 homecooked meals per day. During weight loss maintenance, participants will be given a personalized calorie goal calculated from the Mifflin-St. Jeor [33] equation to sustain their reduced body weight.

Physical activity (PA) is gradually increased through a guided home-based program that incorporates lifestyle activity and overall steps as well as planned exercise minutes. Participants will be guided to gradually increase their PA over the first 12 weeks to 225–250 min/week of moderate intensity activity, consistent with national guidelines for weight loss. If a participant is already exercising regularly, or if a participant has mobility limitations, the lifestyle coach works with the participant to customize goals and activity types based on their current activity and/or mobility level.

*Local Care.* Local Care consists of quarterly PCP visits that follow a 5A's (Assess; Advise; Agree; Assist; Arrange) model, consistent with practice guidelines and the Medical Intensive Behavior Therapy (IBT) benefit [34,35]. Standardized participant hand-outs will be provided to address core elements of obesity treatment guidelines including nutrition and physical activity goal setting and tracking. Local Care handouts cover the same nutrition and physical activity guidelines as the lifestyle intervention in the Team Care arm. Local Care participants also receive a Fitbit and smart scale and will be instructed to use the MyNetDiary app. In addition, the study team assists Local Care clinics in developing a hand-out of local community resources to share with participants.

### 2.9 PCP training

PCP training is designed to be pragmatic so it is feasible with the amount of time busy clinicians may take away from clinical care, based on training experience from RE-POWER [36]. PCP training begins with an initial 4 h of Continuing Medical Education, held separately for each of the four cohorts of practices, to cover guideline-based competencies for obesity treatment [37] (see Table 3). Subsequently, a separate arm-specific training is held at each practice prior to deployment of the intervention. During the intervention period monthly noon-time webinars intend to be offered to provide case consultation with an obesity medicine specialist, collaborative learning, and to cover a variety of “hot topics.” Separate monthly webinars plan to be offered for each arm. In the Team Care arm, lifestyle coaches will join monthly webinars to enhance case discussion and team-based communication.

**Table 3:** PCP training domains and topics.

Domain	Topics
Core knowledge	Obesity as a disease, critical periods, co-morbidities, contextual factors
Behavioral treatment	Guidelines for diet, PA, and behavior change, tracking tools, leveraging local resources
Patient communication	Non-judgmental language, active listening, autonomy support, reducing weight bias
Medication management	Medications causing weight gain, weight loss pharmacotherapy options
Bariatric surgery	Indications, patient education, referral options
Preventing weight regain	Behavioral, psychological, and metabolic factors, extended care, problem-solving
Telemedicine; team-based care	Shared vision, role clarity, accountability, effective communication, visit protocol visits

### 2.10 Outcome measures

Primary, secondary, and tertiary outcome data will be collected across baseline, 6, 12, and 18 months shown in Table 4.

**Table 4:** Primary, secondary, and tertiary outcome measures.

	Milestone			
	BL	6M	12M	18M
**Primary**				
Weight (kg)	X	X	X	X
**Secondary**				
Demographics	X			
Medical, Weight Loss History	X			
Diet quality (REAPS)	X	X	X	X
Physical Activity (MAQ)	X	X	X	X
General Quality of Life (SF-12)	X	X	X	X
Impact of Weight on Quality of Life (IWQOL-L)	X	X	X	X
**Tertiary**				
Mental Health (PHQ-8)	X	X		X
Pain Interference and Intensity (PROMIS-Pain)	X	X		X
Sleep Quality (PSQI)	X	X		X
Patient Activation (PAM-13)	X	X		X
Medical Management of Obesity (PACIC-5A)	X	X		X
Attitudes on Medical Weight Management	X			X
Clinical Management of Weight		X	X	X
Program Satisfaction and Experience of Care		X		X

#### 2.10.1 Primary outcome measure

Weight is collected with the participant fasting and in light clothing, during remote study visits at the participant's home using a study provided Fitbit Aria Air smart scale. Participant will place the scale on a flat solid surface away from walls and in the view of their camera. Participants then weigh themselves twice allowing the scale to zero out between measurements, if the two measurements are not within 0.5 pounds of each other a third measurement is taken.

#### 2.10.2 Secondary outcome measures

Survey measures intend to be collected online unless participants prefer a hard copy by mail (estimated less than 5 % of participants). Demographic, medical and weight loss history will also be collected. Diet quality is assessed via the Rapid Eating Assessment for Participant--Shortened Version (REAPS) [38] which includes 13 items assessing eating and food preparation habits and readiness for dietary change. The Modifiable Activity Questionnaire (MAQ) is used to measure leisure-time physical activity and non-occupational screen time over the past 7 days [39,40]. General health-related quality of life (QoL) is assessed with the SF-12 [41] and obesity-specific QoL is assessed with the Impact of Weight on Quality of Life-Lite (IWQOL-L) measure, with 5 subscales that assess physical function, self-esteem, sexual life, public distress, and work [[42], [43], [44]].

#### 2.10.3 Tertiary outcome measures

Mental health is assessed using the Patient Health Questionnaire-8 (PHQ-8). The PHQ-8 is an 8-item, reliable and valid criteria-based measure of depression severity [45,46]. Pain is assessed using the 4-item pain interference and 1-item pain intensity measure from the Patient Reported Outcomes Measurement Information System-29 (PROMIS-29) [47] that has been shown to have reliability and validity in both general and clinical U.S. populations [48,49]. Sleep quality is assessed with the Pittsburg Sleep Quality Index (PSQI), a 10-item measures that assesses sleep timing and disturbances over the past month [50] and distinguishes between sleep quality in individuals with obesity/metabolic syndrome and those who are of normal weight [51].

#### 2.10.4 Medical treatment process outcomes

The Patient Activation Measure (PAM-13) [52] will evaluate the degree to which the intervention, including the lifestyle coach's involvement in team visits, improves patient engagement, i.e., ability to be involved in treatment, collaborate with providers, and engage in health-promoting behaviors. Team-based care for activating chronic disease self-management and exercise has been shown to improve PAM-13 scores, and a difference of ≥5 points is considered meaningful [53,54]. Treatment process measures related to medical management of obesity is captured using a modified version of the Patient Assessment of Chronic Illness Care (PACIC-5A) measure for obesity treatment in primary care [55]. Participant's past use, perceived safety, and perceived effectiveness of various weight loss methods will be assessed. Survey items are derived from a previous survey that measured perceived safety and efficacy of weight loss methods in patients with obesity [56].

Participants will also assess the helpfulness of their PCP in their weight management using a single item measure that has been associated with greater weight loss in prior primary care-based trials [17]. All medications will be collected at baseline using a structured interview tool where patients gather and report all medications, a method that has resulted in 95 % accuracy [57]. At follow up timepoints, patients will self-report medications for weight loss and/or diabetes via survey, allowing us to identify prescriptions for new weight loss medications. Satisfaction with clinic services and overall satisfaction is measured by items derived from the Integrated Care Performance measure recommendations from the Center for Health Care Strategies [58]. Additionally, counseling satisfaction and group counseling cohesion is measured for patients in the team care arm using items derived from measures previously developed for behavioral weight loss interventions [59,60].

#### 2.10.5 Exploratory measures

The RE-AIM evaluation framework [23] is used to explore the potential population-level impact and generalizability of the intervention in real-world clinical settings. To Measure reach, patient participation rates will be calculated using the registry lists as the denominator and representativeness of enrolled participants versus non-participants are compared based on baseline BMI, age, rurality, and sex. Adoption, implementation, and maintenance at the clinic level will be explored through mixed methods including study-specific surveys and focus groups with participating PCPs. To explore the relative importance of place and context for impacting outcomes, multiple participant-level and spatial-level measures will be collected. Participant-level contextual measures will be collected at baseline. Spatial level measures will be captured from available public data based on participants’ home addresses and include social disadvantage indicators from the American Community Survey [61], food dessert indicators from the USDA Food Access Research Atlas [62], and county-level prevalence of obesity, physical inactivity based on County Health Rankings [63].

### 2.11 Data management and statistical analysis

#### 2.11.1 Sample size

As little as 3–5 % weight loss is clinically meaningful [64]. In RE-POWER, a clinic group-based intervention showed 4.3 % loss at 24 months, and we expect a larger mean effect from the additional medical management component. To determine sample size, we set the type I error at 0.05, intraclass correlation coefficient at 0.02 as was observed in RE-POWER, and 35 participants per clinic. With 16 clinics, the trial will have 85 % power to detect a net between-group treatment effect of 3 % (SD = 8).

#### 2.11.2 Missing data

Based on our prior history using a similar retention plan, we expect to retain at least 85 % of participants at the final 18-month data collection visit, with a goal of 90 %. We will evaluate the missing data pattern for differential loss to follow-up based on treatment arm. In the absence of differential loss to follow-up, the primary analysis described above will use restricted maximum likelihood, and no participants will be deleted from analysis due to missing data. We will conduct a sensitivity analysis to evaluate the sensitivity of the results including only participants who have no missing data.

#### 2.11.3 Statistical analysis for primary aim

Percent weight loss at 18 months will be compared between the treatments. Hierarchical linear mixed models will be used to examine the group differences, accounting for the correlation between participants from the same clinic and for the correlation of measures from the same participants.

#### 2.11.4 Secondary and exploratory aims

Each secondary outcome at 18 months will be compared across arms using separate hierarchical linear mixed model in a similar strategy as the primary aim, except for the proportion achieving clinical cut points of 5 % and 10 % weight loss. These will be compared across arms usings generalized linear mixed models.

To evaluate the impact of pharmacotherapy and bariatric surgery on the between-arm difference in % weight loss, we will add receipt of pharmacotherapy and bariatric surgery to the primary aim to estimate the influence of each on % weight loss beyond other aspects of the intervention. We will use generalized linear mixed models to estimate the proportion who begin pharmacotherapy or undergo bariatric surgery, adjusting for baseline covariates (age, sex, race, education, BMI, insurance, co-morbid conditions), and then multiply the proportion by the effect on % weight loss to calculate impact for each arm, using bootstrapping to derive confidence intervals. We will also conduct a sensitivity analysis on the primary analysis, excluding participants who receive one or both.

Mental health, pain, sleep quality and other medical treatment process outcomes will be compared across treatment arms at 6 and 18 months using separate mixed models in a similar strategy as the primary and secondary aims. Each RE-AIM measure will be compared across treatment arms using descriptive statistics.

To explore the associations between participant-level and tract/county-level contextual measures and % weight loss, we will use hierarchical linear models including variables with the strongest bivariate associations (p < 0.10).

#### 2.11.5 Potential confounders

For all statistical models, sensitivity analyses will be performed using the models above but also adjusting for potential confounders among the baseline variables identified as varying across arms based on their standardized differences.

## 3 Discussion

It is paramount that obesity treatment is offered in rural primary care, especially to reach those who have severe obesity or co-morbid medical conditions because they are at the greatest risk for developing obesity-related chronic disease and certain cancers. Primary care has the potential to fill a major gap in access to evidence-based weight control programs in rural communities. Primary care practices must capitalize on guideline-based medical management concurrent with lifestyle change for weight loss as it is essential to address co-morbid medical conditions, evaluate medications, and explore options for anti-obesity medications or bariatric surgery. With the expanded capacity for home-based telemedicine visits due to COVID-19, novel models for integrating intensive group-based behavioral obesity interventions in rural primary care while addressing cost and workforce barriers have emerged. This study is unique in that the findings will compare the effectiveness of a collaborative model that combines the benefits of group-based treatment, interprofessional team-based care, and participant activation with the lifestyle coach before, during, and after quarterly clinic visits to standard quarterly primary care visits. This model of care capitalizes on the importance of medical management, access to local support and resources, and knowledge of the community. Few trials have examined inter-professional team-based clinical care models for obesity treatment involving at least two health care providers who work collaboratively toward shared goals. The results may warrant a new standard of care for obesity treatment in rural primary care practices.

## CRediT authorship contribution statement

**Alexandra R. Brown:** Writing – original draft. **Edward F. Ellerbeck:** Writing – review & editing, Investigation. **Debra K. Sullivan:** Writing – review & editing, Investigation. **Eve-Lynn Nelson:** Writing – review & editing. **Jennifer R. Klemp:** Writing – review & editing. **Byron J. Gajewski:** Writing – review & editing. **Jarron Michael Saint Onge:** Writing – review & editing. **Christie A. Befort:** Writing – review & editing.

## Declaration of competing interest

The authors declare that they have no known competing financial interests or personal relationships that could have appeared to influence the work reported in this paper.

## Data Availability

No data was used for the research described in the article.
